# A population-based study of cognitive impairment in socially vulnerable adults in Argentina. The Matanza Riachuelo study preliminary results

**DOI:** 10.1590/S1980-57642014DN84000006

**Published:** 2014

**Authors:** Leonardo Bartoloni, Graciela Blatt, Iván Insua, Mariano Furman, María Agustina González, Bárbara Hermann, Mariana Kesselman, Alicia Massautis, Alejandra Reinado, Patricia Senas, Claudia Yavitz, Horacio Lejarraga, Fernando Nunes, Raúl Luciano Arizaga, Ricardo F Allegri

**Affiliations:** 1 ACUMAR-Autoridad de Cuenca Matanza Riachuelo (Dirección General de Salud Ambiental). Ciudad de Buenos Aires. República Argentina.; 2 Hospital Dr. Abel Zubizarreta (Centro de investigaciones cognitivas). Ciudad de Buenos Aires. República Argentina.; 3 Centro de Memoria y Envejecimiento, Instituto de Investigaciones Neurológica (FLENI), Ciudad de Buenos Aires. República Argentina.; 4 PRONADIAL (Programa Nacional de Datos, Docencia e Investigación en Alzheimer y otros Trastornos Cognitivos). Departamento de Salud Pública. Facultad de Medicina. Universidad de Buenos Aires. Argentina.

**Keywords:** cognitive impairment, dementia, prevalence, social vulnerable population, Argentina

## Abstract

Population aging has taken place intensively worldwide, even in developing countries. These countries have population groups with low resources and basic unmet needs that are frequently omitted from epidemiological studies.

**Objective:**

The purpose of this study was to determine the prevalence of cognitive impairment (CI) and dementia in an economic and socially vulnerable population from Argentina.

**Methods:**

A door-to-door observational population-based survey among adults over 60 years of cognitive impairment and dementia in the social vulnerable area of the Matanza Riachuelo Basin, in the suburban area of Buenos Aires, Argentina was conducted. Trained psychologists interviewed subjects and a proxy informant. A standardized protocol including a socio-demographic questionnaire, the Mini-Mental State Examination, the Geriatric Depression Scale and a functional inventory for IADL and ADL was administered. Diagnoses were divided into three general categories: normal cognitive function, cognitive impairment-no dementia (CIND) and dementia.

**Results and Conclusions:**

A total of 2437 elderly persons were assessed, of which 73.6% fulfilled inclusion criteria. The prevalence of CI among those over 60 was 26.4% (18.1% CIND and 8.3% dementia) with higher prevalence of dementia in younger individuals than rates reported in developed counties, probably due to low control of vascular risk factors. This information can help inform health public decisions in the generation of programs and plans for the prevention, diagnosis and treatment of cognitive impairment in this type of socially vulnerable population.

## Abstract (pt)

O envelhecimento da população idosa tem aumentado mundialmente, mesmo em países em desenvolvimento. Estes países têm grupos populacionais de poucos recursos, com necessidades básicas não satisfatórias, frequentemente omitidas nos estudos epidemiológicos.

**Objetivo:**

O propósito deste estudo foi determinar a prevalência de comprometimento cognitivo (CC) e demência em uma população socioeconomicamente vulnerável na Argentina.

**Métodos:**

Foi realizada pesquisa observacional de base populacional porta-a-porta entre adultos acima de 60 anos numa área de vulnerabilidade social em Matanza Riachuelo, no subúrbio de Buenos Aires, Argentina. Psicólogos treinados entrevistaram o sujeito e um acompanhante. Um protocolo estandardizado que incluiu um questionário sociodemográfico, o MEEM, a escala de depressão geriátrica e um inventário funcional para ABVD e AIVD foram administrados. Os diagnósticos foram divididos em três categorias: função cognitiva normal, comprometimento cognitivo sem demência (CCSD) e demência.

**Resultados:**

Foram avaliados um total de 2437 de idosos, dos quais 73,6% preencheram os critérios de inclusão. A prevalência de CC entre aqueles acima de 60 anos foi de 26,4% (18,1% CCSD e 8,3% de demência) com maiores prevalência de demência em indivíduos mais jovens do que os reportados em países desenvolvidos, provavelmente devido a controle inadequado dos fatores de risco vasculares.

**Conclusão:**

Esta informação pode contribuir para tomada de decisões em saúde pública para geração de programas e planos que apontem para a prevenção, diagnóstico e tratamento de CC neste tipo de população em vulnerabilidade social.

## INTRODUCTION

Numerous advances in science in general, and in medicine in particular, over recent decades have led to an increase in the elderly population (over 60 years) causing an exponential growth in age-dependent pathologies such as dementia.^1^

The process of demographic transition (aging population) has occurred more quickly than was expected a few years ago, even in developing countries.^2^ This has had a major impact on public health and the economy leading to reviews of health, social and economic policies in developed and developing countries.

Within this aging group, many subjects seek assistance for "memory impairment". This complaint is very common in the course of normal aging, but is also characteristic of the early stages of dementia.^3^

After 60 years, some of the most common conditions are "cognitive disorders" in their different clinical forms (mild cognitive impairment and dementia). According to the WHO, these disorders are the leading cause of disability in this age group.^4-6^

The study of dementia is lower than for chronic non-communicable diseases such as cardiovascular disease and cancer. Demographic transition has been faster this millennium in low- and middle-income countries (LMIC) than last century in high-income countries (HIC)(World Population Prospects, 2003). The prevalence is higher in LMIC, attaining 7.1 % in South America.^7-9^

Scant data are available on the prevalence of cognitive impairment in Argentina because of the death of epidemiological studies on prevalence or incidence of dementia. The data available is partial, incomplete and scarce. Melcom et al. reported the first data in 1996.^10^ The authors collected the information from death certificates in the city of Junin within the province of Buenos Aires, Argentina, where the rates for dementia were 110.3/100,000, with significant increases beyond the age of 75.^10^ The limiting factors of the study were that the results were based on reports by doctors made at the time of death of patients, and it was unusual to describe dementia as a cause of death during that period.

in 2004, Pages Larraya et al. found cognitive impairment in 23% of subjects over 60 years but the study was conducted with institutionalized subjects in nursing homes.^11^

Arizaga et al. carried out a survey involving demographic data, risk factors and Geriatric Depression Scale and MMSE scores in a population over 60 in Cañuelas, 50 km from Buenos Aires, finding a cognitive impairment prevalence of 22.3 % (individuals with 22 points or less on the MMSE).^12^ As part of the 10/66 ADI Project, Arizaga et al. conducted in the Prevalence Phase of the project both in a rural area (Cañuelas) and in Buenos Aires city, Argentina. Although, the study could not be completed and the partial results will be published in the near future.

According to these partial data and the result of the latest national population survey in the year 2010 (INDEC, National Institute for Statistics and Census),^13^ there were approximately 1,279,294 individuals with cognitive impairment and 614,061 with dementia.

In conclusion, to date, no epidemiological study has been conducted in Argentina with all necessary information (prevalence and incidence of cognitive impairment or dementia) for planning public health strategies.

Census data released in 2010 by the INDEC^13^ showed that about 12.5 percent of the population lived in slums with basic unmet needs. This indicates that 4.9 million out of the 40.1 million population of the country resided in areas of irregular occupation characterized by lack of public services or urbanization.^13^

**Figure 1:**
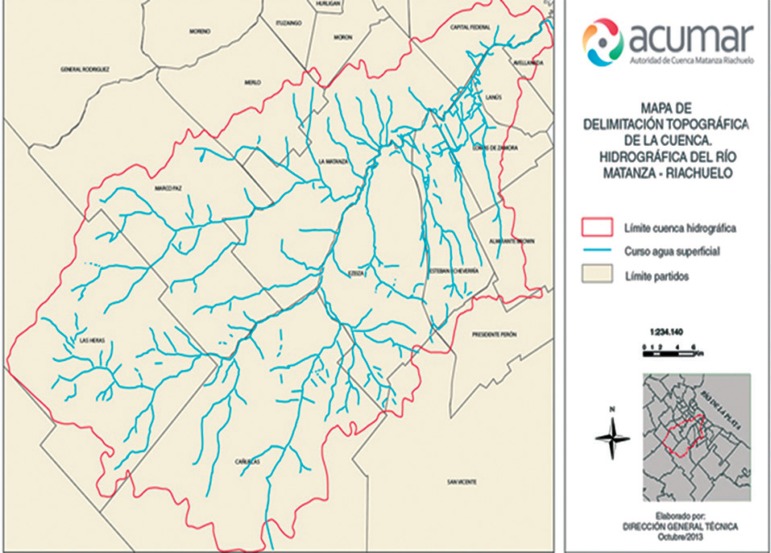
Map of study area of Matanza and Riachuelo basin (Buenos Aires - Argentina).

The basin of the Matanza and Riachuelo rivers (see Figure 1) is a heavily populated urban informal settlement covering 2200 km^2^ in the southeast of Buenos Aires city and several cities surrounding the metropolis and home to the majority of the slums. These slums have 8,212,953 inhabitants that lack reliable sanitation services, supply of clean water, reliable electricity, law enforcement teams, and have a low socioeconomic level, poverty and social vulnerability.^14^

On July 8, 2008 ACUMAR (Authority of the Matanza Riachuelo region) was created to run a program with the articulation of public policies and infrastructure, which in turn can improve the quality of life for residents of the basin, rebuild all the components of the environment (water, air and soil) and finally prevent unnecessary and predictable damage.^15^

With the objective of improving the quality of life of the inhabitants of the Matanza Riachuelo, a HEP(Health Emergency Plan) was put in place to implement the prevention and control processes that tend to minimize the impact of various threats detected on the health of the basin population, which is run by the Department of Environmental Health, under the ACUMAR.^15^

A component for health in Aging was established in the ACUMAR in early 2012, with the collaboration of the National Policy for Older Persons, Ministry of Social Development of the Nation (DINAPAM).^15^ The main objective was to describe the status of the population of older adults aged 60 or over in the Matanza Riachuelo basin region, from the perspective of cognitive functions, and its relationship with the particular demographic and environmental factors of this region.

The purpose of this study was to determine the prevalence of cognitive impairment (CI) and dementia in an economic and socially vulnerable population of Argentina.

## METHODS

**Study design and setting**. The Matanza-Riachuelo Study is an on-going, observational, descriptive, cross-sectional door-to-door population-based survey among all residents over 60 years in the Matanza Riachuelo basin, which is a 2200 km^2^ area covering part of the south of Buenos Aires city and the neighbouring counties in the southeast of Buenos Aires City (Lanus, Avellaneda, Lomas de Zamora, Esteban Echeverria, La Matanza, Ezeiza, Canning, Almirante Brown, Moron, Merlo, Marcos Paz, Presidente Peron, San Vicente and General Las Heras).^13^

A total of 8,212,953 people reside (2010) in this slum area in conditions of abject poverty. Slums arise and persist for a combination of demographic, social, economic and political reasons. Common causes include rapid rural-to-urban migration from others parts of the country and other South America countries involving people of all ages including the aged.

**Population**. Seven slums in the Matanza Riachuelo area (Villa flammable, Wilde, Acuba, Villa 21-24, Villa 26, Villa Garden and Villa Lamadrid) were selected for study based on the worst conditions of poverty.^13^

To increase participation in this study a first approach describing the programme was made. The study protocol was explained and discussed in meetings with local councilors and local health authorities and the resident population.

The following exclusion criteria were applied:

[1] elderly not present for three consecutive attempts at home visits;
[2] individuals who refused to participate in the study;
[3] persons willing to participate but with difficulties answering or interpreting the initial survey due to sensory deficits (blindness, deafness, etc.);
[4] persons diagnosed with developmental delay;
[5] subjects not speaking Spanish fluently;
[6] illiterate;
[7] institutionalized patients; and
[8] itinerant patients with a temporary home address (expected to be at this address for less than six months).

**Survey**. A door-to door population-based survey was carried out between June 2012 and September 2013. Because of the age, fragility and vulnerability of the population investigated, a screening phase was performed at participants' homes and a second phase of diagnosis for positive participants in a truck prepared for this assessment. When present, a proxy informant (usually a family member) was also interviewed.

The screening phase was performed by a psychologist specifically trained for the survey to collect the socio-demographic information about lifestyle, habits, medical history and health status from the subject and the informant. During the same visit, a test battery including the Argentine adaptation of the Mini-Mental State Examination (MMSE),^16-19^ the Spanish version of the Geriatric Depression Scale (GDS)^20^ and a comprehensive questionnaire for functional impairment for instrumental activities of daily living (IADL) and basic activities of daily living (ADL)^21^ was administered.

**Measurements**. The MMSE in its original version^14^ and its translation and adaptation to Spanish form the Rio de la Plata (Argentina) was used. The MMSE cut-off was established based on the validated version,^18,19^ which is 24 points for a senior younger than 75 years and 5 or more years of education. The cut-off was 22 for elders aged 75 years or more and less than 5 years of education.^18,19^

Depression features were assessed with the Spanish version of the Geriatric Depression Scale (GDS)^20^ and functional assessment was done by a comprehensive questionnaire of functional impairment for instrumental activities of daily living (IADL) and basic activities of daily living (ADL).^21^

**Criteria used to determine positive results**. Based on these MMSE results, the population was divided into a normal group and a subgroup with probable cognitive impairment.

For the sample population, probable dementia was defined, in addition to scoring below the MMSE cut-off, based on functional status as a measure of patients ability to perform activities of daily living independently (person must be dependent in at least two functional areas, leading to interference in IADL and/or ADL) and impairment in the past two years (representing a decline from the previous level of functioning).

Diagnoses were divided into three general categories: normal cognitive function, cognitive impairment-no dementia (CIND) and dementia according the DSM-IV criteria.

**Training and quality control**. A psychologist, psychiatrist and neuropsychologist were recruited locally and followed a training course on each aspect of the research every 6 months given by RFA and LB. All difficulties were discussed with the study coordinator. All questionnaires and scales were reviewed and discussed by the study coordinator (M), RFA and LB. During the first month of the survey, feasibility was tested and the procedure optimized. Several changes were implemented in the MMSE for this population with social vulnerability:

[1] the term "street" was changed to "neighbourhood", because in the place of assessment, there are no structured homes and streets;
[2] a phrase was changed in the repetition test (the term raspberries was cut), being "flan has strawberries" largely because subjects could not repeat the phrase in full, for not knowing the meaning or terminology of raspberries;
[3] in the writing test, the wording was changed, being "Write a comment/phrase/or sentence with a meaning"; and
[4] the attention test by "numerical subtraction" was removed due to level of education and difficulty in understanding the example.

**Ethical issues**. Study procedures were in accordance with the Rules of ICH Good Clinical Practice,^22^ the revised declaration of Helsinki 1964, with its amendments,^23^ and the legislation of the national regulatory authority, ANMAT^24^ of the City of Buenos Aires^25^ and the Province of Buenos Aires.^26^ References have been taken from international ethical standards, such as the Nuremberg Code,^27^ Declaration of Helsinki,^23^ as amended; as well as the Universal Declaration on Human Genome and Human Rights adopted by the General Conference of UNESCO, 11 November 1997.^28^

**Statistical analysis**. The study sample was tabulated in percentages (Nominal variables) and mean and standard deviations (numerical variables) of sociodemographic characteristic, medical history, clinical features, as well as cognitive, mood and functional profile. The data were analysed using the SPSS/PC version 18.0 statistical package (SPSS Inc., Chicago, IL, USA)

## RESULTS

**Figure 2:**
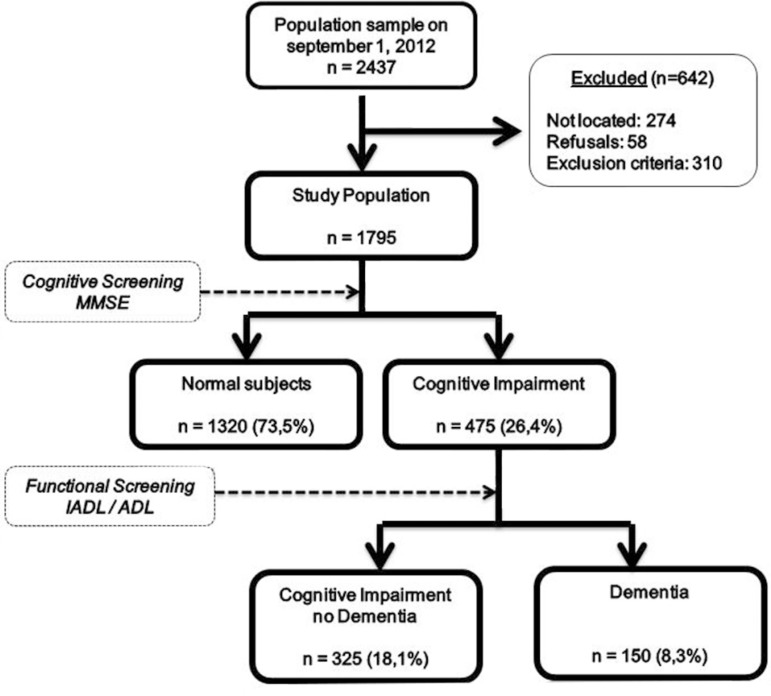
Flow chart of Matanza Riachuelo study.

**Study population**. Figure 2 shows the flow diagram of the study, 274 of the 2437 individuals over 60 contacted in the study (11.2%) were absent at the time of being visited by the interviewers, 58 (2.3%) refused to participate and 296 (12.1%) were excluded (others exclusion criteria). The final sample was 1795 (73.6%) individuals to be evaluated.

The demographic characteristics and risk factors of the populations assessed are reported in Table 1.

**Table 1:** Demographic data.

Nº		1795
Age (years)		68.4 (10.8)
Sex (%women)		55.8%
Education (years)		5.6 (3.3)
MMSE (total score)		25.2 (4.3)
Medical coverage	Not covered	25%
	PAMI	65%
	Other	10%
Risk factors	Hypertension	43.5%
	Diabetes mellitus	12.6%
	Dyslipidemia	17.0%

Note: age, education and MMSE expressed as mean (standard deviations).

**Figure 3:**
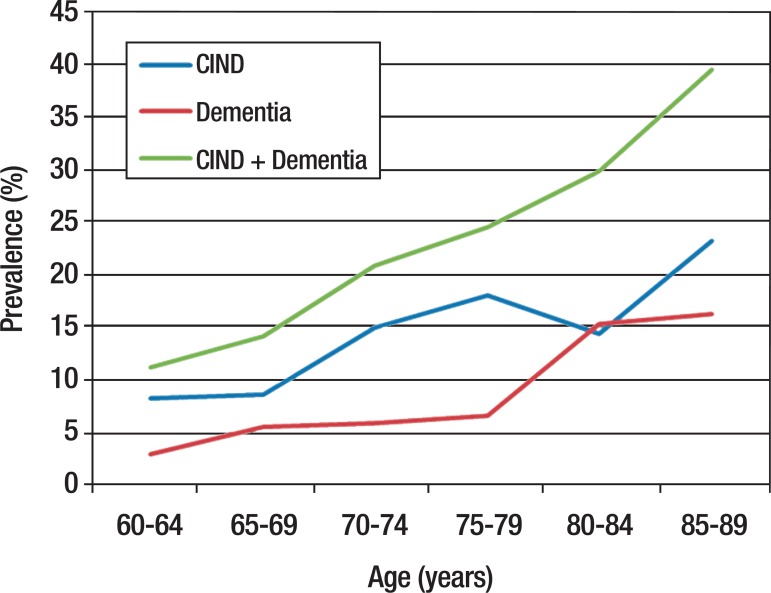
Prevalence of cognitive impairment and dementia according to age.

Applying the cut-off scores used for the present study, 475 individuals (26.4%) were detected as possible cases of cognitive impairment and 1320 subjects (73.5%) as cognitively normal individuals. Based on their functional status, 15% of individuals were considered CIND and 8,3% dementia. Table 2 and Figure 3 show the results according to age group and sex.

**Table 2:** Age- and sex-specific prevalence ratios of dementia.

	Total			Men			Women	
	nº	%		nº	%		nº	%
60-64 years	15/510	2.9		5/201	2.4		10/308	3.2
65-69 years	24/435	5.5		9/200	4.5		15/234	6.4
70-74 years	18/307	5.8		9/141	6.3		9/166	5.4
75-79 years	13/200	6.5		7/90	7.7		6/110	5.4
80-84 years	16/104	15.3		7/38	18.4		9/66	13.3
>85 years	11/58	18.9		4/15	26.6		7/34	20.5

## DISCUSSION

The data available on the epidemiology of dementia in socially vulnerable adults is relatively scarce. Epidemiological studies in these populations pose a methodological and logistical challenge. Such studies require rigorous training and the adaptation of conventional tools for use in this population. It is for this reason that 27% were excluded. Among the main causes of exclusion were the peculiar characteristics of the catchment area in which older adults were evaluated, absence of the elderly at home at the time of evaluation, refusal to participate, and exclusion criteria precluding application of the assessment tools proposed (such as illiteracy, sensory deficits, language disabilities, etc.).

The observed prevalence of 15% for CIND and 8.3% for dementia implies a total of 23.3% of all people aged over 60 with CI. Meaningful comparisons with other studies conducted in Argentina are therefore difficult^10,11^ while global results were similar to those found by Arizaga et al.^12^(CI of 22.3%).

Results from the present study have demonstrated that our survey findings are similar to data reported in other Latin American studies.^8^ The global prevalence rate for dementia in this study was 8,3% while the rate in systematic reviews of prevalence studies in LA was 7.1%,^12^ and most studies from European countries, Japan and the USA have reported prevalence rates of between 5.5% and 9.0% in those aged 65 or over.^31,32^

A limitation of the study was that the preliminary results were from the first part of the Matanza Riachuelo study in which possible cases of cognitive impairment or dementia were detected that will later need to be verified in the second phase through detailed neuropsychological assessment.

Another finding was a higher prevalence of dementia in younger individuals in this type of social vulnerable population of 2.9% in those aged 60 to 64 years and 5.5% in those 65 to 69 years. One possible explanation is the limited access to primary care services during life, the low educational level (mean 5,6 years) and the high level of vascular risk factors. Similar results reported by Nitrini et al. in LA studies were higher than figures observed in developed countries.^8^

The presence of modifiable risk factors (through primary prevention) can change the presentation and progression of different cognitive pictures. It is noteworthy that, despite the particular social situation of the population studied, a starting point for the promotion of plans could be to prevent the advance and disability in older adults of cognitive impairment and dementias.

To conclude, the Matanza-Riachuelo study is one of the largest prospective population-based studies specifically focused on the research of cognitive decline and dementia in a socially vulnerable population. Prevalence of dementia was similar to that reported in LA and other developed countries, being higher in relatively young subjects compared to developed countries, probably due to the characteristics of this population.
